# The Role of Oxytocin in Alzheimer’s Disease and Its Relationship with Social Interaction

**DOI:** 10.3390/cells12202426

**Published:** 2023-10-10

**Authors:** Junpei Takahashi, Daisuke Yamada, Wakana Nagano, Akiyoshi Saitoh

**Affiliations:** Laboratory of Pharmacology, Faculty of Pharmaceutical Sciences, Tokyo University of Science, 2641 Yamazaki, Noda 278-8510, Chiba, Japan; 3b21706@ed.tus.ac.jp (J.T.); yamadada@rs.tus.ac.jp (D.Y.); 3b23547@ed.tus.ac.jp (W.N.)

**Keywords:** Alzheimer’s disease, amyloid beta, dementia, cognitive impairment, learning, memory, social isolation, oxytocin

## Abstract

Alzheimer’s disease (AD)—the most common cause of dementia in the elderly—is characterized by progressive memory loss and β-amyloid protein (Aβ) accumulation in the brain. Recently, loneliness was found to be a high risk factor for AD, and social isolation has become a major cause of AD. AD. Oxytocin (OXT), the main hormone involved in social bonding, has been implicated in social interactions, notably in building trust and relationships. Moreover, social isolation or social enrichment modulates the activation of neurons related to OXT. Recently, we reported that OXT reverses learning and memory impairment in AD animal models. Based on the limited number of studies currently available, OXT might be a therapeutic target for AD. Further studies are necessary in order to better understand the role of oxytocin in AD. In this review, we described the relationships between OXT, AD, and social interaction.

## 1. Introduction

Alzheimer’s disease (AD), first reported by Alois Alzheimer in 1907, is recognized by the World Health Organization (WHO) as a global public health priority [1]. Although the pathogenesis and treatment of AD have been widely reported, disease-modifying treatments are still lacking [2]. Patients with AD mainly present with cognitive impairment and behavioral and psychological symptoms of dementia, such as anxiety, depression, and aggression. AD pathogenesis involves the accumulation of amyloid beta (Aβ) peptides and the overexpression of phosphorylated tau in the brain. These Aβ peptides are soluble, 39–42 amino acid-long metabolic fragments formed by the cleavage of the amyloid precursor protein (APP). In AD, the Aβ peptides aggregate to form insoluble amyloid fibrils in senile plaques and cerebral vasculature in the brain [3,4]. However, recent studies have shown that the Aβ oligomers are more neurotoxic than the Aβ fibrils [5]. These Aβ oligomers are aggregates of 2–30 Aβ polymers. Small Aβ oligomeric aggregates of 2–12 polymers are highly neurotoxic and can eventually form large oligomers, fibrils, and plaque [6]. The dimeric Aβ oligomers isolated from the brains of patients with AD can inhibit long-term potentiation (LTP) and enhance long-term depression in the hippocampus by using electrophysiology. Shanker et al. suggested that these dimers are the smallest units of synaptotoxic species [7]. However, higher-order oligomers, including aggregates of 12–24 polymers, have been shown to be highly neurotoxic [8,9]. Despite the differing opinions regarding this, the Aβ oligomers are integral to AD pathogenesis and are thus therapeutic targets for AD. Further, the overexpression of phosphorylated tau causes AD development. Normally, tau functions as a stable microtubule for axons [10,11]. However, hyperphosphorylated tau forms neurofibrillary tangles (NFTs) in neurons, causing apoptosis [12,13]. Thus, Aβ oligomers, senile plaques, and NFTs are important features of AD patients’ brains, and the removal of Aβ has recently gained popularity as a potential therapeutic strategy for AD [14,15].

Oxytocin (OXT) is a peptide hormone synthesized in the paraventricular hypothalamic nucleus (PVN) and the supraoptic nucleus (SON). It is released peripherally from the posterior pituitary into the blood and exerts a peripheral effect that facilitates parturition and lactation [16,17]. In 1953, Du Vigneaud et al., revealed the structure of OXT and synthesized it for the first time [18,19]. Thirty years later, Buijs et al., detected OXT fibers in the brain regions and showed that they acted as neurotransmitters/neuromodulators [20]. The neuronic projections and receptors of OXT are widely distributed in the regions responsible for cognitive functions, such as the hippocampus, perirhinal, entorhinal cortices, and supramammillary nucleus [18,19]. The OXT receptor, a seven-transmembrane G protein-coupled receptor, is capable of binding to either G_i/o_ or G_q/11_ proteins and activates a set of signaling cascades, such as the Mitogen-activated Protein Kinase (MAPK), Protein Kinase C (PKC), Phospholipase C (PLC), Ca^2+^/calmodulin-dependent protein kinase (CaMK), and cAMP-responsive element binding protein (CREB) pathways [19,21]. Most OXT receptors in the hippocampal dentate gyrus are present in GABAergic neurons [22], whereas in the hippocampal CA1, 2, and 3, OXT receptors are expressed in glutamatergic neurons [22,23].

The role of OXT in cognitive function and the detailed mechanisms of its action have been reported [24]. Notably, the intracerebroventricular (ICV) administration of OXT enhances long-term spatial memory through increased CREB phosphorylation in the hippocampus [24]. Moreover, OXT perfusion in hippocampal slices from nulliparous female and male rats enhances the late phase of long-term potentiation (L-LTP) [21]. Furthermore, the OXT-induced enhancement of L-LTP is inhibited by OXT receptor antagonists and downstream partner inhibitors [21]. Overall, OXT enhances cognitive functions via various signaling cascades.

OXT also regulates anxiety, depression, and social and cognitive behaviors [25]. In particular, OXT-deficient mice exhibited high levels of anxiety-related behavior in an elevated plus maze test, which was reversed through OXT administration [26]. The intraperitoneal or intranasal administration of OXT suppresses lipopolysaccharide (LPS)-induced anxiety-related behavior in mice following a decrease in plasma corticosterone levels [27]. OXT rescues stress-induced memory and learning impairments. Lee et al., reported that uncontrollable stress, such as restraint and tail shocks, impairs spatial memory and that hippocampal LTP can be reversed by OXT administration [28]. Furthermore, acute stressful stimuli induce OXT release from the amygdala [29]. Overall, stressful stimuli can activate the OXT neurons. Moreover, OXT buffers stress-induced mental and physical impairments.

Based on the limited number of studies currently available, OXT might be a therapeutic target for AD. Further studies are necessary in order to better understand the role of oxytocin in AD. Here, we reviewed publications in the existing literature that describe the relationships between OXT and various stressors, placing special emphasis on studies on the associations between OXT and social isolation.

## 2. Loneliness Aids AD Development

In 2007, Wilson et al., hypothesized that loneliness increases the risk of developing AD [30]. They examined this hypothesis using longitudinal clinicopathologic study data— Rush Memory and Aging Project (MAP) data—to enumerate the risk factors of geriatric chronic conditions. They uniformly evaluated the participants of this study (at baseline and annually thereafter for up to 4 years) for loneliness, the clinical classification of dementia and AD, and detailed cognitive functioning. A total of 823 older persons without dementia served as controls in the study. The loneliness of participants was assessed annually using a modified version of the de Jong Gierveld Loneliness Scale [31] with a five-item scale at baseline. The five items included the following: “I experience a general sense of emptiness”, “I miss having people around”, “I feel like I don’t have enough friends”, “I often feel abandoned”, and “I miss having a really good friend”. During follow-up, 76 subjects developed clinical AD. The data were fitted using a hazards model that controlled for age, sex, and level of educational achievement. They found that the risk of clinical AD increased by approximately 51% for each point increase on the loneliness scale (relative risk, 1.51; 95% confidence interval, 1.06–2.14). Thus, a person with a high degree of loneliness (score 3.2) was approximately 2.1 times more likely to develop clinical AD during follow-up compared to a person with a low degree of loneliness (score 1.4) [30].

Nancy et al., verified if amyloid burden (a putative in vivo biomarker of AD) is associated with social interaction. Their study included 79 participants (43 women and 36 men), the mean age of which was 76 years. They measured cortical amyloid burden (using PiB-PET) and loneliness (using UCLA-3 loneliness score, a commonly used measure of loneliness). They found that amyloid burden was associated with loneliness. Lonely individuals exhibited 7.5 times more amyloid positivity compared to socially active individuals. They hypothesized that loneliness might occur in association with elevated amyloid burden [32].

Interestingly, family size is a risk factor of AD [33]. In order to correlate household size to dementia mortality, You et al., conducted a retrospective cross-sectional study comparing dementia mortality rates in 183 member states of the WHO and the data of each respective country on household size. Although household size was negatively and moderately strongly correlated with dementia mortality, the results suggested that the larger the household, the lower the dementia mortality. Thus, a large household protects against dementia mortality [34]. Shen et al., investigated the association of social isolation and loneliness with dementia incidence and explored the potential underlying neurobiological mechanisms. They found that social isolation was associated with a 1.26-fold increased risk of dementia. Furthermore, social isolation decreased gray matter volumes in the temporal, frontal, and hippocampal areas [33].

Animal studies suggest that social isolation aids AD progression. Peterman et al., examined whether social isolation stress accelerates the onset of AD in 5×FAD mice. The effect of 2–3 months isolation, beginning at 2 months of age, on cognitive functioning was assessed using contextual fear conditioning experiments. Compared to group-housing, isolation significantly decreased the freezing time in both 5×FAD+ and 5×FAD- mice. However, the isolation significantly increased amyloid β plaque in 5×FAD+ mice compared to 5×FAD- mice. Furthermore, three-month isolation significantly increased BACE1 expression in 5×FAD+ mice compared to 5×FAD- mice. These results suggest that social isolation attenuates cognitive behavior irrespective of genotype. Furthermore, social isolation accelerated the amyloid β plaque burden and BACE1 expression in 5×FAD mice [35].

Ren et al., found that compared to group-housing, six weeks of social isolation increased the expression of hyperphosphorylated tau in the hippocampus of middle-aged rats (8 months of age) [36]. Thus, social isolation may be a risk factor of AD. Interestingly, social enrichment reversed the Aβ-induced impairment of short- and long-term objective memory and social recognitive memory [37].

Social isolation may be one of the triggers leading to AD development. Social isolation affects the endocrine system (e.g., the hypothalamic–pituitary–adrenal (HPA axis) and OXT neurons). These changes may be involved in the development of AD. The pathogenic alterations of AD occur early in the brain; i.e., they occur before the appearance of clinical symptoms [38]. Therefore, it is expected that constant communication with others can prevent the onset of AD. Recently, due to the COVID-19 outbreak, people experienced increased social isolation [39]. In addition, COVID-19 accelerated cognitive decline in elderly patients with dementia [40] and potentially increased the risk of cognitive impairment [41]. Therefore, the number of patients with AD is expected to increase worldwide because of the increase in social isolation and the COVID-19-mediated aggravation of neurological symptoms.

## 3. Social Environment Affects OXT Neuron Activation

### 3.1. Social Enrichment Increases the Activity and Secretion of OXT

Odendaal et al., found that, in humans and dogs, serum OXT, β-endorphin, prolactin, and dopamine levels increased after 5–25 min of social interaction, while serum cortisol levels decreased. They suggested that the increase in OXT levels helps mediate social bonding [42]. Kosfeld et al., proposed that, in humans, OXT increases trust and that the intranasal administration of OXT increases the likelihood of entrusting money to a stranger [43]. In rats, social enrichment has a greater effect on increasing plasma OXT and extending telomere length compared to standard social interaction [44]. Neal et al., found that rats housed in a socially enriched environment had increased OXT immunoreactivity in PVN and SON compared to normally housed or socially isolated rats. In addition, compared to normally housed rats, plasma OXT levels were also increased in rats housed in a socially enriched environment [45]. These reports suggested that OXT is involved in social interaction and bonding and that social environment affects the activity and secretion of OXT.

### 3.2. Effects of Social Isolation on OXT Activity and Secretion

Are social isolation and OXT neurons activity associated? Indeed, social isolation and OXT neurons activity affect each other. Karelina et al., reported that OXT mRNA and OXT receptor gene expressions were decreased in adult mice following 1 week of social isolations [46]. Tanaka et al., also immunohistochemically examined changes in central OXT neural activity induced by post-weaning isolation. They found that compared to group-reared rats and unlike male rats, female mice who had been socially isolated for 14 days showed a reduced number of OXT cells in the PVN [47]. Thus, social isolation might decrease the activity and secretion of OXT.

Contrarily, Elias et al., reported elevated OXT mRNA in the PVN and a decreased expression of OXT receptors in the bed nucleus of the stria terminalis and nucleus accumbens in female rats subjected to social isolation post-weaning, and this contrasted with the results obtained for the study’s male rats [48]. Grippo et al., found that social isolation for 60 days increased plasma OXT, AVP, and CORT levels in female prairie voles. In addition, OXT- and CORT-immunoreactive cells in the PVN increased in socially isolated prairie voles compared to those in group housing [49]. Thus, social isolation may increase the activity and secretion of OXT. OXT can suppress the activity of the HPA axis [50,51,52,53]. Thus, OXT may inhibit social isolation-associated stress responses. In fact, OXT reverses social isolation-induced behavioral disorders in rodents. Han et al., reported that subjecting mice to social isolation for 5 weeks increased their anxiety-like behaviors in an open field test and depression-like behaviors in forced swimming and sucrose preference tests.

In summary, social isolation affects OXT neuron activity. However, the results from different studies cannot be directly compared, as OXT levels were measured differently in each one. In fact, there is discrepancy in the reported associations between OXT neuron activity and social isolation. Thus, further research involving the use of improved OXT measurement methods is necessary to clarify the relationship between OXT and social isolation.

Despite inconclusive literature data, we hypothesize that social isolation negatively affects OXT neurons. In addition, we speculate that, under normal conditions, OXT neurons are activated to suppress HPA axis activation, which is induced by social isolation stress. Thus, an imbalance in OXT neuron activity and HPA axis interactions may lead to AD development. Decreases in OXT neuron activity induced by social isolation stress may aid AD development.

## 4. OXT Is a Potential Therapeutic Target for AD

### 4.1. OXT Reverses The Impairment of Learning and Memory in AD-Model Mice

Recently, we reported for the first time in the literature that OXT reversed Aβ-induced cognitive impairment. These results have been successfully replicated. Reports have shown that OXT can recover cognitive function impairment and Aβ deposition in AD rodent models. However, the role of OXT as a potential therapeutic target for AD has not been explored yet.

OXT rescues impaired learning and memory in AD-model mice. We recently reported that OXT reverts Aβ_25–35_-induced cognitive function impairment in rodents [54,55] and LTP in the mouse hippocampus. Furthermore, these effects are mediated by the OXT receptor. Pretreatment with L-368,899 (a selective oxytocin receptor antagonist) antagonizes the OXT-mediated reversal of impaired learning and memory in AD-model mice. Additionally, pretreatment with U0126 (a selective MEK/ERK inhibitor) and NASPM (a selective antagonist of Ca^2+^-permeable AMPA receptors) blocked the rescuing effect of OXT on the Aβ_25–35_-induced impairment of LTP. Thus, ERK phosphorylation and Ca^2+^-permeable AMPA receptors are involved in this effect of OXT [54]. In addition, we showed that the ICV administration of Aβ_25–35_ to mice impairs spatial working memory, as demonstrated by spontaneous alternations in the Y-maze test and spatial memory, as demonstrated by the latency to the platform in the Morris Water Maze test. Interestingly, pretreatment with L-368,899 inhibited the OXT-mediated rescue of impaired spatial working memory in these mice. Thus, we propose that central nervous system (CNS) OXT may be a target of future dementia therapeutics.

However, ICV administration of OXT is very invasive and impractical for potential clinical application. Moreover, peptides are characterized by poor blood–brain barrier (BBB) permeability. Intranasal administration is a clinically applicable technique used to deliver proteins and peptides into the CNS. For this purpose, we prepared OXT derivative-containing cell-penetrating peptides (CPPs) and a penetration-accelerating sequence (PAS). Intranasal administration is generally adopted to deliver proteins and peptides into the brain via the olfactory neuronal distribution pathway in the cribriform plate, resulting in direct nose-to-brain drug distribution [56]. CPPs can enhance cellular uptake through micropinocytosis, while PAS can promote the escape from the endosomal vesicles and each cell. Therefore, the PAS-CPP peptide derivatives can effectively translocate from the nasal mucosa to the neurons inside the brain [57].

The intranasal administration of the OXT derivative improved memory function, but the intranasal administration of native OXT did not (Y-maze test). Moreover, using the fluorescein isothiocyanate-labeled OXT, we ascertained that the intranasally administered OXT derivative was delivered into the brain; the fluorescence of the intranasally administered labeled-OXT derivative was distributed throughout the mouse brain. Thus, the OXT derivative can be efficiently delivered from the nose to the brain to improve murine memory [55]. El-Ganainy et al., also reported that intranasal OXT attenuates the spatial memory deficits (Morris Water Maze tests) observed in aluminum chloride AD-model rats (oral aluminum chloride (100 mg/kg) daily for 8 consecutive weeks) [58]. Furthermore, in AD-model rats, OXT inhibited the increase in acetylcholinesterase protein levels; Aβ_1–42_, ERK1/2, and GSK3β activities; and tau phosphorylation. It also inhibited hippocampus CA1 morphological changes. This report, for the first time in the literature, elucidated that OXT has a potential therapeutic effect on AD and acts by suppressing acetylcholinesterase activity, hippocampal Aβ deposition, and tau levels [58]. The specific activation of CNS OXT receptors via the intravenous injection of OXT-loaded angiopep-2-modified chitosan nanogels that can cross the BBB and specifically bind to OXTR reversed the impairment of spatial memory in APP/PS1 mice (Morris Water Maze tests). Furthermore, using magnetic resonance imaging, Ye et al., showed that the hippocampal CA1 region volume was reduced in the APP/PS1 mice compared to the control mice. More excitingly, treatment with OXT nanogels alleviated the decrease in CA1 volume in these APP/PS1 mice [59]. Western blotting analysis showed that the administration of the OXT nanogel inhibited the observed increase in the hippocampal phosphorylation of ERK and p38 in the APP/PS1 mice. Furthermore, the OXT nanogel inhibited increases in the inflammatory signals iNOS, COX-2, TNF-α, and IL-1β in APP/PS1 mice. Moreover, the OXT nanogel suppressed the increased Aβ deposition seen in APP/PS1 mice. Thus, OXT-induced inhibition of inflammatory signaling cascades might inhibit Aβ deposition and delay neuronal apoptosis. Moreover, Ye et al., investigated the safety of OXT nanogels and found that various biosafety markers, including white blood cells, hemoglobin, platelets, aminotransferase, serum albumin, creatinine, red blood cells, aspartate transaminase, blood urea nitrogen, and total bilirubin, were similar between the OXT nanogel-treated and untreated groups, indicating that the OXT nanogel is safe to use [59]. Recently, an immunohistology study showed that OXT immunoreactivity was decreased in APP/PS1 mice. Maria et al., also reported that the exposure of hypothalamic slices to Aβ oligomers and the ICV administration of Aβ oligomers reduced OXT mRNA expression in the hypothalamus. Furthermore, studies have reported that the intranasal administration of OXT can recover social and nonsocial (objective and spatial memory) memory in APP/PS1 mice, as verified via the use of intruder and novel object recognition and Morris Water Maze tests. Moreover, immunohistology analysis results showed that OXT administration decreased the expression of Aβ deposition, Iba1 and CD68, indicating that OXT attenuates memory deficits and brain inflammation [60].

### 4.2. The Activity of OXT Neurons Is Altered in AD Patients

OXT is implicated in AD pathology. Despite clinical evidence linking OXT to AD pathology, there is lack of consensus about the exact modulation of brain OXT concentrations in AD pathology. Using voxel-based morphometry and MRI Cloud analysis, Petekkaya et al., measured differences in medial lobe structures and neuropeptide levels in AD patients diagnosed with mild AD and healthy individuals. They found that the AD patients had a lower-right hippocampus volume. Interestingly, plasma OXT concentration was lower in AD patients than the healthy controls. Furthermore, they conducted a correlation analysis. It was observed that, in patients with AD, the right parahippocampal gyrus volume was positively correlated with an increase in the plasma OXT concentration and negatively correlated with the OXT signal value. Thus, in patients with AD, OXT concentration and OXT signal values were altered in the brain areas involved in cognitive functioning [61]. Zou et al., used gene expression profiles to explore markers associated with AD and cerebral small vessel disease (CSVD) in patients with AD and CSVD [62]. Differential analysis was performed between AD and mild cognitive impairment or CSVD progression and CSVD no-progression. In both datasets, 146 differentially expressed genes with the same expression direction, all of which are included in the OXT signal pathway, were identified. The OXT signal cascade might be a marker for AD and CSVD progression [62]. Lardenoije et al., also explored AD biomarkers by investigating changes in DNA methylation [63]. They found epigenetic differences in AD patients compared to controls in the middle temporal gyrus, and the *OXT* gene was methylated in AD patients. Moreover, they compared the blood methylome of converters to AD and nonconverters at the preclinical stage. The methylation of the *OXT* gene in blood in converters to AD were found to be associated with the methylation of the *OXT* gene in the middle temporal gyrus in AD patients. They suggested that the methylation of the *OXT* gene may be a target for future studies on early biomarkers and novel therapeutic strategies in AD [63].

In contrast, using immunoreactivity, Mazurek et al., reported that OXT concentration was increased by 33% in the hippocampus and temporal cortex of Alzheimer brains compared to healthy brains. Mazurek proposed that OXT levels may contribute to memory disturbances associated with AD [64]. However, the mean profile area of OXT neurons did not change significantly with increasing age. Fliers et al., investigated the interaction between aging and OXT cells in PVN and SON. Brain samples were obtained from 32 patients aged between 10 and 93 years, including 3 patients with Alzheimer-type senile dementia. Although only a few cases of AD were studied, they reported that there might be a decrease in the activation of OXT neurons rather than degeneration in the brains of AD patients [65]. In 1986, through using a radioimmunoassay, Murray et al., measured OXT, vasopressin (AVP), somatostatin, and β-endorphin levels in the cerebrospinal fluid (CSF) of AD patients and healthy individuals of all ages. They found that AVP and somatostatin levels in AD patients were lower compared to that in healthy individuals of all ages. However, OXT and β-endorphin levels were similar in AD patients and healthy individuals of all ages [66]. Furthermore, in 1991, Wierda et al., compared the number of OXT neurons in PVN in healthy individuals (15–90 years old) and AD patients (46–97 years old). They found that the number OXT neurons in PVN was similar in healthy individuals and AD patients. In addition, no correlation was observed between the number of OXT neurons and age or brain weight, suggesting that OXT level and OXT neurons are not involved in AD [67].

Although studies showing the relationship between AD patients and OXT are quite contradictory, understanding *OXTR* polymorphisms might clarify this. Phenotypes are controlled by various single nucleotide polymorphisms (SNPs). rs53576 and rs2254298, located within intron 3 of the OXTR gene, are two of the most frequently reported OXTR SNPs [68,69]. These SNPs influence social abilities in children with autism spectrum disorder (ASD) or attention deficit hyperactivity disorder (ADHD) [69]. The authors of [69] showed that, while the OXTR SNPs (rs53576 and rs2254298) were associated with an increased severity of social deficit in ASD and ADHD, rs237887 and rs13316193 were not [69]. Kumsta et al., reported that OXTR SNPs were responsible for the difference in OXT sensitivity through using a pharmacogenetic approach [70]. They found that the intranasal administration of OXT enhanced emotion recognition performance in the TTCGG haplotype, comprising many SNPs (rs237917–rs2268498–rs4564970–rs237897–rs2268495–rs53576-), compared to placebo. However, the CCGAGA carriers showed the opposite pattern [70]. These reports indicate that the OXTR SNPs might contribute to differences in human behavior (social ability and cognitive) and OXT sensitivity. Therefore, differences in OXTR SNPs might be involved in AD pathogenesis; hence, investigating these polymorphisms might elucidate the association between AD pathogenesis and OXT. Moreover, the DNA methylation of OXTR also affects human behavior. Increased OXTR DNA methylation is associated with social, cognitive, and emotional impairments, while decreased OXTR DNA methylation is linked to mood swings and anxiety disorders [71]. Therefore, DNA methylation of OXT and OXTR might also participate in AD pathogenesis. Furthermore, differences in polymorphisms and DNA methylation can be potential AD biomarkers.

Taken together, OXT alleviates cognitive impairment in AD-model animals. It is also safe to use, making it an ideal therapeutic target for treating AD. However, the precise effect of OXT on AD symptoms is still unclear. Therefore, further research is needed to clarify this. Moreover, clinical studies linking OXT and AD have also been inconclusive. As different OXT measurement methods have been used in many different studies, the results of these studies cannot be mutually compared. Therefore, further studies involving improved OXT measurement methods, well-documented study subjects, and the use of OXTR polymorphisms must be conducted to clarify the relationship between OXT and AD. Recently, a clinical trial was initiated to investigate the safety of intranasal OXT on AD patients (jRCTs061200039, Okayama, Japan), indicating that OXT might be a valid therapeutic candidate for AD. Therefore, this review will help clinicians and scientists who are engaged in AD research.

## 5. Conclusions

Recently, due to the COVID-19 outbreak, people are reeling from the effects of increased social isolation. This social isolation is expected to increase the number of AD patients with aggravated neurological symptoms. Therefore, it is becoming increasingly important to investigate the relationship between social interactions and AD. This review has summarized the associations between AD, OXT, and social environment. Social isolation alters the activity of OXT neurons and leads to the development of AD; however, the details underlying this are unknown. This review underscores the need for increased socialization in managing AD. Importantly, OXT rescues cognitive impairment and Aβ accumulation in AD-model animals. Thus, OXT is a potential therapeutic target in the treatment of AD (Table 1 and Table 2).

## Figures and Tables

**Table 1:** Summary of interactions between OXT and social environment in animal experiments (upper row). Social environment affects the activity of OXT neuron. Summary of social environment and AD (lower row). Social isolation affects AD development and progression.

Loneliness Aides AD Development			
References	Species	Subjects/Model	Results
[30]	Persons	A total of 823 older persons with no dementia.	A person with a high degree of loneliness was about 2.1 times more likely to develop clinical AD, compared to a person with a low degree of loneliness.
[32]	Male and female persons	A mean age of 76 years.	The amyloid burden was associated with loneliness. The amyloid-positive group was found to be 7.5 times more lonely individuals than those who were not lonely.
[33]	Male and female persons	A total of 462,619 persons (mean age at baseline 57.0 years).	Social isolation was associated with 1.26-fold increased risk of dementia.
[34]	Male and female persons	Dementia specific mortality rates of the 183 member states of World Health Organization were calculated and matched with the respective country data on household size.	The larger household, the less the dementia mortality.
[35]	5 × FAD mice	The isolation for 2 or 3 months began at 2 months of age.	Social isolation in 5×FAD mice accelerated the burden of amyloid β plaque and BACE1 expression.
[36]	Male Sprague Dawley rats	Social isolation for 6weeks in middle-aged rats (8-month age).	Social isolation increased in expression of hyperphosphorylated Tau in hippocampus compared to group housing.
Social Environment Affects Activation of OXT Neurons			
References	Species	Model	Results
[44]	Male and female Wistar rats	After weaning at PND 21, rats live at standard housing or social housing.	Social enrichment increases plasma OXT and extends telomere length.
[46]	Male C57/BL6 mice	Mice housed either individually or with an ovariectomized female for a period of 1 week prior to surgery and throughout the reperfusion period.	OXT mRNA gene and OXT receptor gene were decreased following social isolation for 1week in adult mice.
[47]	Rats	Social isolation for about 14 days was performed on male or female mice at 24–28 days of age.	OXT cells in PVN of isolated female rats were decreased compared to group housing, not but male rats.
[48]	Male and female Wistar rats	Isolation rats were housed in a single rat cage at PND21-PND72.	Elevated OXT mRNA in the PVN and decreased expression of OXT receptors in the bed nucleus of the stria terminalis and nucleus accumbens of females by post-weaning social isolation but not in males.
[49]	Female prairie voles	Twenty adult female prairie voles were exposed to either 60 days of social isolation or paired housing.	OXT- and CORT-immunoreactive cells in the PVN increased in social isolated prairie voles compared to group housing.

**Table 2:** Summary of interactions between OXT and AD in animal (upper row) or human studies (lower row). The administration of OXT reversed cognitive dysfunction in AD-model mice. The results are, however, inconclusive.

Animal Studies			
References	Species	AD-Model	Results
[54]	Male ddy mice	Perfusion of Aβ_25–35_ in the hippocampus slice	The perfusion of OXT reverses Aβ-induced impairment of hippocampal long-term potentiation via OXT receptors, ERK phosphorylation and Ca^2+^-permeable AMPA receptors.
[55]	Male ddy mice	The ICV administration of Aβ_25–35_	The ICV administration of OXT recovered Aβ_25–35_ -induced impairment of spatial working memory and spatial reference memory by Y-maze and MWM.
[58]	Female Sprague Dawley rat	A daily oral dose of aluminum chloride (100 mg/kg) for 8 consecutive weeks	The intranasal OXT reversed impairment of spatial memory, suppressed AL-increased protein levels and restored morphological changes in the hippocampus.
[59]	Female APP/PS1 mice	Transgenic mouse: amyloid precursor protein (Mo/HuAPP695swe) and a mutant human presenilin 1 (PS1-dE9)	The intravenously injection of OXT nanogel reversed impairment of spatial memory by MWM. OXT nanogel inhibited inflammatory signaling cascades to delay Aβ deposition and neuronal apoptosis.
[60]	Male APP/PS1 mice	Transgenic mouse: amyloid precursor protein (Mo/HuAPP695swe) and a mutant human presenilin 1 (PS1-dE9)	Intranasal administration OXT recovered social and objective and spatial memory in APP/PS1 mice. OXT showed decreased in expression of Aβ deposition, Iba1 and CD68 by immunohistology.
Activity of OXT Neurons is Altered in AD Patients			
References	Gender	Subjects	Results
[61]	Male and female	Patients diagnosed with mild AD Normal people (72.9 ± 4.5 years of age)	AD patient group had a lower right hippocampus volume and plasma OXT concentration than the control group.
[62]	Gene expression profiles of AD and CSVD were downloaded from the gene expression omnibus (GEO) database. gene expression profiles of blood samples from 145 AD subjects, 80 MCI subjects, and 104 healthy controls.		DEGs include in OXT signal pathway are identified between AD and mild cognitive impairment (MCI) or CSVD progression and CSVD no-progression.
[63]	Male and female	Brain tissue was obtained from 80 individuals aged above 80 years. Blood DNA was obtained from 99 individuals aged above 75 years.	OXT gene in the middle temporal gyrus was methylated in AD patients. And the methylation of OXT gene in blood in converters to AD was found to be associated with the methylation of OXT gene in the middle temporal gyrus in AD patients.
[64]	Male and female	The postmortem brain tissue from 12 cases (62–90 years old) of Alzheimer’s disease and 13 controls (36–87 years old).	OXT concentration was increased 33% in the hippocampus and temporal cortex of Alzheimer brains comparable to control group.
[65]	Male and female	32 patients at 10 to 93 years of age including 3 individuals with senile dementia of the Alzheimer type.	The size of OXT cells did not show any significant changes with increasing age.
[66]	Male	Patients with AD (67 ± 2.3 years of age) Normal elderly (68 ± 2.7 years of age) Normal young (25 ± 0.6 years of age)	The OXT level in cerebrospinal fluid was similar in AD patients and healthy individuals.
[67]	Male and female	Patients with AD (46 to 97 years of age) Normal people (68 ± 2.7 years of age)	The number OXT neurons in PVN was similar in healthy individuals and AD patients.

## Data Availability

Not applicable.
